# A Munc18-1 mutant mimicking phosphorylation by Down Syndrome-related kinase Dyrk1a supports normal synaptic transmission and promotes recovery after intense activity

**DOI:** 10.1038/s41598-020-59757-y

**Published:** 2020-02-21

**Authors:** Jessica Classen, Ingrid Saarloos, Marieke Meijer, Patrick F. Sullivan, Matthijs Verhage

**Affiliations:** 1 Department of Functional Genomics, Center for Neurogenomics and Cognitive Research, VU University, 1081 HV Amsterdam, The Netherlands; 2 Department of Medical Epidemiology and Biostatistics, Karolinska Institutet, Nobels väg 12A, PO Box 281, 171 77 Stockholm, Sweden; 3 Departments of Genetics and Psychiatry, University of North Carolina at Chapel Hill, Chapel Hill, North Carolina USA

**Keywords:** Molecular neuroscience, Neuronal physiology

## Abstract

Phosphorylation of Munc18-1 (*Stxbp1*), a presynaptic organizer of synaptic vesicle fusion, is a powerful mechanism to regulate synaptic strength. Munc18-1 is a proposed substrate for the Down Syndrome-related kinase dual-specificity tyrosine phosphorylation-regulate kinase 1a (*Dyrk1a*) and mutations in both genes cause intellectual disability. However, the functional consequences of Dyrk1a-dependent phosphorylation of Munc18-1 for synapse function are unknown. Here, we show that the proposed Munc18-1 phosphorylation site, T479, is among the highly constrained phosphorylation sites in the coding regions of the gene and is also located within a larger constrained coding region. We confirm that Dyrk1a phosphorylates Munc18-1 at T479. Patch-clamp physiology in conditional null mutant hippocampal neurons expressing Cre and either wildtype, or mutants mimicking or preventing phosphorylation, revealed that synaptic transmission is similar among the three groups: frequency/amplitude of mEPSCs, evoked EPSCs, paired pulse plasticity, rundown kinetics upon intense activity and the readily releasable pool. However, synapses expressing the phosphomimic mutant responded to intense activity with more pronounced facilitation. These data indicate that Dyrk1a-dependent Munc18-1 phosphorylation has a minor impact on synaptic transmission, only after intense activity, and that the role of genetic variation in both genes in intellectual disability may be through different mechanisms.

## Introduction

Post-translational modification of synaptic proteins is a powerful way to regulate synaptic strength. Phosphorylation is probably the most well-studied mechanism of regulation of synaptic protein function. Many synaptic proteins have been identified in large-scale phospho-proteomics screens^1–4^, although the kinase and the function of the phosphorylation are often unknown. Presynaptic proteins (e.g. Synapsin, SNAP25, synaptotagmins, and Munc18) are phosphorylated by abundant (not neuro-specific) kinases like protein kinase C (PKC) and protein kinase A (PKA) and these modifications impact on synaptic transmission in a variety of ways (see review^5^). However, many more (potential) phosphorylation sites in synaptic proteins are reported, for which the impact is unknown.

Munc18-1 is a major regulator of synaptic transmission and among the best validated presynaptic phosphorylation targets. Loss of Munc18-1 in neurons leads to a loss of neurotransmitter release and cell death *in vivo*^6^ and *in vitro*^7^. *Munc18-1 null* neurons survive for 4 days in culture but are subsequently unable to support viability^7^. The MUNC-18 gene in human is *STXBP1*, and exonic mutations in *STXBP1* have been reported to cause *STXBP1*-encephalopathy, characterized by severe intellectual disability (ID), spasms, and epilepsy^8–11^. Many of these clinical symptoms are also observed in *Stxbp1* mouse models of *STXBP1*-encephalopathy^12^.

Several potential phosphorylation sites of Munc18-1 have been found^1^ or predicted^13^. To date, all confirmed phosphorylation sites have major impacts on Munc18-1 function: protein kinase C (PKC)^14–16^, Src^17^ and ERK^18^. Munc18-1 is also reported as a target for Dyrk1a, a kinase involved in Down syndrome which phosphorylates Munc18-1 at threonine 479 (T479)^19^. The functional impact of this modification is unknown.

The dual-specificity tyrosine phosphorylation-regulate kinase (Dyrk) family are serine/threonine kinases that regulate many cellular processes (see review^20^). Dyrk1a is located in the nucleus and cytoplasm of neurons in the mouse cerebellar cortex^21^, plasma membrane in synaptosomal fractions^22^, and in the human brain^23^, and is involved in neuronal differentiation^24–26^, regulating the balance between proliferation and apoptosis^27^, dendrite and spine outgrowth^28^, and tau phosphorylation^23,29,30^. The human *DYRK1A* gene is located in the Down syndrome critical region and its levels are important in the pathogenic mechanism^31–33^. Furthermore, mutations in *Dyrk1a* are linked to other forms of ID^9^, similar to mutations in STXBP1/Munc18-1^11^. Mouse models for Dyrk1a and STXBP1 haploinsufficiency show similar autistic-like features and seizures^12,34^, and full deletion of both Dyrk1a and Munc18-1 is embryonically lethal^6,35^. Dyrk1a knockout mice are lethal because phosphorylation of Hip-1 blocks neuronal cell death in neural progenitor cells and promotes neurite outgrowth^36^. Hence, these similarities between Dyrk1a and Munc18-1 strengthen the idea that the phosphorylation of Munc18-1 by Dyrk1a might be important for synapse function and contributes to an explanation for the severe ID caused by mutations in the two genes.

Therefore, we hypothesized that phosphorylation of Munc18-1 by Dyrk1a regulates the role of Munc18-1 in neuronal survival, synaptic transmission, and plasticity. To investigate this, we used patch clamp electrophysiology in single hippocampal neurons. We found that mimicking phosphorylation of Munc18-1 by Dyrk1a, and comparing it to a non-phosphorylatable Munc18-1 and a wildtype, did not affect basic synaptic transmission or short-term plasticity parameters such as release probability and the size of the readily releasable pool. However, the recovery of the readily releasable pool was increased by mimicking Dyrk1a-dependent phosphorylation. In conclusion, mimicking phosphorylation of Munc18-1 by Dyrk1a is not essential for synaptic transmission but promotes recovery.

## Results

### The proposed *Stxbp1* T479 target site for Dyrk1a is among the highly constrained phosphorylation sites and is also located within a larger constrained coding region

**Figure 1:**
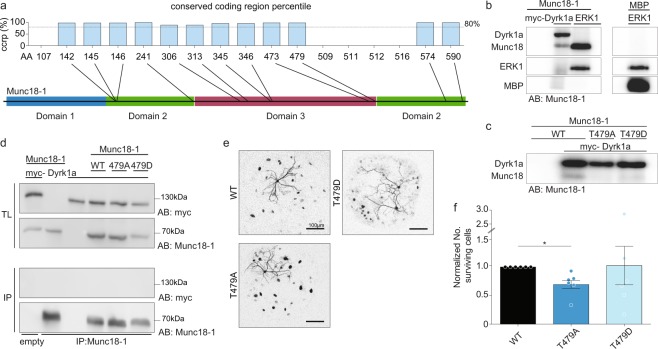
Dyrk1a phosphorylates Munc18-1. (**a**) Predicted and confirmed phosphorylation sites of Munc18-1. Sites with a conserved coding region percentile (ccrp) above 80% are considered notable (*upper*). Positions of the notable predicted phosphorylation sites are mapped onto a schematic representation of Munc18-1 (*lower*). (ccrp is only reported for notable sites). (**b**) *In vitro* kinase assay. Gel of immunoprecipitation (IP) of HEK293T cell lysate expressing Munc18-1 or MBP. Precipitate was incubated with kinase buffer (containing myc-Dyrk1a, ERK or no kinase) in the presence of 25 μM [γ-32P]-ATP. The first lane is an IP not incubated with a kinase. Gel was cropped for clarity (full length gel available in Supplementary Fig. S1). (**c**) *In vitro* kinase assay. Gel of immunoprecipitation (IP) of HEK293T cell lysate expressing M18_WT_, M18_T479A_ or M18_T479D_ incubated with kinase buffer (containing Dyrk1a or no kinase) in the presence of 25 μM [γ-32P]-ATP. The first lane has no added kinase. Gel was cropped for clarity (full length gel available in Supplementary Fig. S1). (**d**) Cell lysate from HEK293T cells expressing M18_WT_, M18_T479A_ or M18_T479D_ together with or without myc-Dyrk1a were immunoprecipitated (IP) with Munc18-1 antibody (αM18). Dyrk1a was immunoblotted for whether it was immunoprecipitated with Munc18-1 using αmyc (top IP), after which the blot was reblotted for Munc18-1 (bottom IP). Total lysate (TL) is shown for loading control (immunoblotted for αM18 and αmyc at the same time). The blot includes the control for non-specific binding conducted with empty beads (lane 1), and in the absence of Dyrk1a (lane 2) and Munc18-1 (lane 3) for specificity of the interaction. Blots were cropped for clarity (full blot available in Supplementary Fig. S1). (**e**) Example pictures of *munc18 null* neurons rescued with either of the three Munc18-1 constructs, immunostained with MAP2. (**f**) Quantification of surviving *munc18 null* neurons transfected with either M18_WT_, M18_T479A_ or M18_T479D_, normalized to cells expressing M18_WT_, to control for batch to batch variation (M18_WT_: n = 6; M18_T479A_: n = 6; or M18_T479D_: n = 6; Kruskal-Wallis test and Dunn’s multiple comparison test, M18_WT_ versus M18_T479A_ = *P* < 0.05, M18_WT_ versus M18_T479D_ = *P* > 0.05, M18_T479D_ versus M18_T479A_ = *P* > 0.05).

STXBP1 has multiple known phosphorylation sites that were identified in large-scale phosphoproteomic screens looking at *in vivo* phosphorylation of synaptic proteins^1^ and tyrosine phosphorylation sites in the mouse brain^37^ as well as potential phosphorylation sites^13,19^. To prioritize these, we overlaid orthogonal empirical data. Using whole exome sequencing data from 123,136 humans, (PMID 30531870)^38^ we identified exonic regions that were *markedly* intolerant to variation across the whole exome. Genetic variation can be expected to occur virtually everywhere in the genome: if genetic variation is not observed in an exon-sized region in adult humans, this region is constrained and likely subject to strong purifying selection. Given the known criticality of STXBP1 to synaptic function, we wished to prioritize phosphorylation sites that were highly intolerant to exonic mutation: 12 of 18 Stxpb1 phosphorylation sites were found to be highly intolerant to variation (constrained coding region intolerance scores in the 89–99th percentile, Fig. 1a, Supplementary Table S1). The proposed Stxbp1 T479 target site for Dyrk1a is among the highly constrained phosphorylation sites and is also located within a larger constrained coding region (Supplementary Table S2).

### Dyrk1a phosphorylates but does not co-precipitate Munc18-1

Currently, *in vivo* evidence for Dyrk1a phosphorylating Munc18-1 is lacking but *in vitro* experiments suggest that Dyrk1a phosphorylates Munc18-1 at T479^19^. To confirm this, an *in vitro* kinase assay was used, in which isolated rodent Munc18-1 was incubated with purified active Dyrk1a or the validated kinase ERK as positive control^18^ in the presence of [γ-32P]-ATP. We confirmed that active Dyrk1a phosphorylates Munc18-1 (Fig. 1b). Amino acid substitution of the target amino acid T479 to either aspartic acid or alanine prevents phosphorylation of Munc18-1 (Fig. 1c), thereby validating T479 as the Dyrk1a target site. However, using co-immunoprecipitation, no stable interaction between Munc18-1 and Dyrk1a was detected (Fig. 1d). These findings suggest that under our experimental conditions, Dyrk1a transiently interacts with Munc18-1 to phosphorylate the T479 residue.

### Dyrk1a-dependent Munc18-1 site T479 plays a minor role in neuronal survival

Dyrk1a regulates cortical development via many targets^27,29,39,40^. To exclusively investigate the functional impact of Dyrk1a-dependent Munc18-1 phosphorylation, we expressed one of two phosphorylation mutants, replacing the threonine at amino acid 479 either with aspartic acid (M18_T479D_, to mimic the phosphorylated state) or alanine (M18_T479A_, to prevent phosphorylation) in *munc18 null* neurons. Wildtype Munc18-1 (M18_WT_) served as a positive control. *Munc18 null* neurons die within days *in vivo* and *in vitro*^6,7^. Survival of *munc18 null* neurons expressing M18_T479D_ was comparable to M18_WT_ expressing neurons, but higher variability was observed in neurons expressing M18_T479D_. Compared to neurons expressing M18_WT_, survival of M18_T479A_ expressing neurons was reduced by 15% (p < 0.05, Fig. 1f). Hence, the mutant that cannot be phosphorylated at T479 shows a minor reduction in supporting cellular viability.

### Neuronal morphology and Munc18-1 levels are normal in neurons mimicking Dyrk1a- phosphorylation of Munc18-1

**Figure 2:**
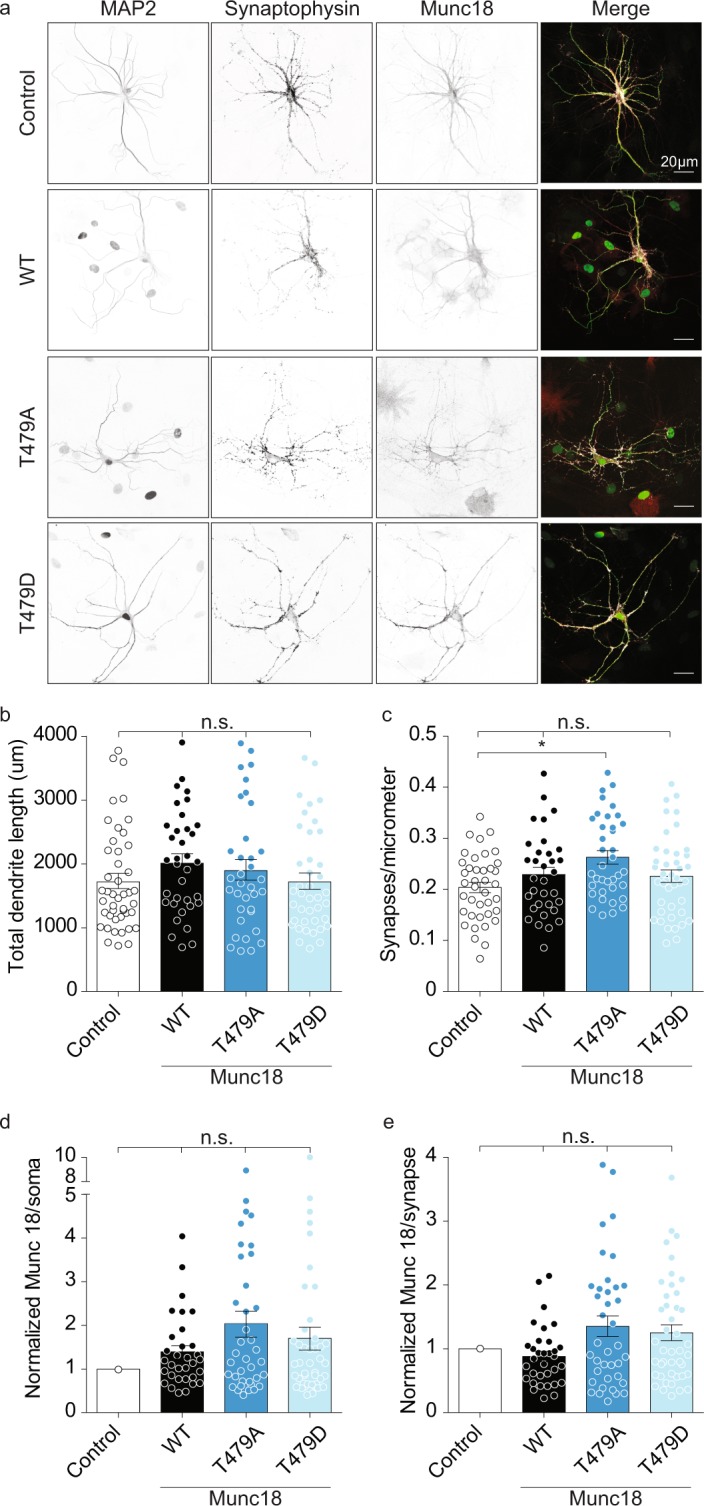
Phosphorylation of Munc18 by Dyrk1a has no effect on neuronal morphology. (**a**) Typical images of *Munc18-1 lox* neurons expressing either M18_WT_, M18_T479A_ or M18_T479D_, together with Cre, or untreated neurons as a control for endogenous levels of Munc18-1. Neurons were stained for MAP2, Synaptophysin, and Munc18-1. (**b**) Total dendrite length (Control: 1720 ± 119.6 µm, n = 45; M18_WT_: 2010 ± 136.6 µm, n = 35; M18_T479A_: 1895 ± 161.4 µm, n = 35; or M18_T479D_: 1716 ± 127 µm, n = 40; all *P* > 0.05). (**c**) Synapses per micrometre (Control: 0.2 ± 0.01, n = 39; M18_WT_: 0.23 ± 0.01, n = 33; M18_T479A_: 0.26 ± 0.01, n = 37; or M18_T479D_: 0.23 ± 0.01, n = 42; all *P* > 0.05). (**d**) Normalized Munc18-1 levels in the soma. Normalized to the average endogenous level of Munc18-1 derived from the control condition (M18_WT_: 1.39 ± 0.15, n = 32; M18_T479A_: 2.04 ± 0.3, n = 37; or M18_T479D_: 1.7 ± 0.26, n = 42; all *P* > 0.05 except Control versus M18_T479A_: *P* < *0.05*). (**e**) Normalized Munc18-1levels per synapse. Normalized to the average endogenous level of Munc18-1 derived from the control condition (M18_WT_: 0.88 ± 0.08, n = 33; M18_T479A_: 1.35 ± 0.16, n = 38; or M18_T479D_: 1.25 ± 0.12, n = 43; all *P* > 0.05). (Statistically tested using the Kruskal-Wallis test and Dunn’s multiple comparison test).

To test whether neuronal development and synapse formation are influenced by Dyrk1a-dependent phosphorylation of Munc18-1, we compared neuronal morphology and synapse development in single hippocampal *Munc18-1 lox* neurons expressing Cre-EGFP and M18_T479A_, M18_T479D_ or M18_WT_ after 15 days *in vitro* (Fig. 2a). Dendrite length was the same between the conditions (Fig. 2b), while neurons expressing M18_T479A_ had about 10% more synapses per micrometre neurite than control neurons, which have not been infected with virus (Fig. 2c).

Munc18-1 levels are known to influence synaptic strength^41^. To control for possible confounding effects of potentially different expression levels in experiments with different phophomutants and to test the effect of Dyrk1a-dependent Munc18-1 phosphorylation on Munc18-1 cellular half-life, we compared the expression levels of M18_T479A_, M18_T479D_ and M18_wt_ using immunocytochemistry. The expression levels of all Munc18-1 variants were similar (Fig. 2d,e). Hence, neuronal morphology, synapse formation and cellular Munc18-1 levels were all similar among neurons expressing wildtype or one of the phosphomutants, aside from some minor variation.

### Synaptic transmission is normal, but synaptic recovery is enhanced in neurons mimicking Dyrk1a-phosphorylation of Munc18-1

**Figure 3:**
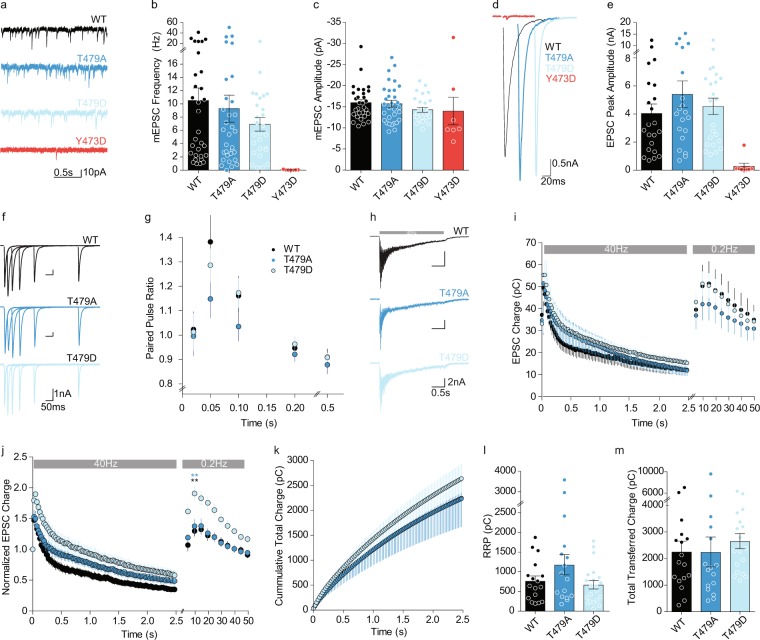
Phosphorylation of Munc18 by Dyrk1a influences recovery from readily releasable pool depletion. (**a**) Typical examples of passive recordings showing mEPSCs. (**b**) mEPSC frequency (M18_WT_: 10.52 ± 1.89 Hz, n = 31; M18_T479A_: 9.29 ± 2.04 Hz, n = 32; M18_T479D_: 6.93 ± 1.03 Hz, n = 27; M18_T473D_: 0.5 ± 0.02 Hz, n = 7; *P* = 0.6823, M18_WT_ versus M18_T479A_ = *P* > 0.05, M18_WT_ versus M18_T479D_ = *P* > 0.05, M18_T479D_ versus M18_T479A_ = *P* > 0.05). (**c**) mEPSC amplitude (M18_WT_:−16 ± 0.72 pA, n = 32; M18_T479A_: −15.73 ± 0.83 pA, n = 32; or M18_T479D_: −14.29 ± 0.58 pA, n = 27; M18_T473D_: −14 ± 3.25 pA, n = 7; *P* = 0.2674, M18_WT_ versus M18_T479A_ = *P* > 0.05, M18_WT_ versus M18_T479D_ = *P* > 0.05, M18_T479D_ versus M18_T479A_ = *P* > 0.05). (**d**) Example traces of evoked EPSCs. (**e**) Evoked EPSC amplitude (M18_WT_: −4.04 ± 0.67 nA, n = 22; M18_T479A_: −5.4 ± 0.95 nA, n = 20; or M18_T479D_: −4.69 ± 0.59 nA, n = 23; M18_T473D_: −0.27 ± 0.21 nA, n = 8; *P* = 0.507, M18_WT_ versus M18_T479A_ = *P* > 0.05, M18_WT_ versus M18_T479D_ = *P* > 0.05, M18_T479D_ versus M18_T479A_ = *P* > 0.05). (**f**) Typical examples of paired pulse traces. Traces from different intervals are superimposed (20 ms, 50 ms, 100 ms, 200 ms, and 500 ms). (**g**) Quantification of the paired pulse ratio for different intervals (M18_WT_: n = 22; M18_T479A_: n = 20; or M18_T479D_: n = 24). (**h**) Typical examples of 40 Hz stimulation train. (**i**) Quantification of EPSC charge during 40 Hz train stimulation. Followed by a 0.2 Hz train stimulation to monitor post-stimulation recovery (M18_WT_: n = 17; M18_T479A_: n = 17; or M18_T479D_: n = 19). (**j**) Normalized EPSC charge quantification during 40 Hz train stimulation and 0.2 Hz. The 0.2 Hz recovery train EPSC charge is normalized to the first EPSC of the 40 Hz train stimulation (M18_WT_: n = 17; M18_T479A_: n = 17; or M18_T479D_: n = 18; 0.2 Hz 2^nd^ pulse, *P* = 0.0004, M18_WT_ versus M18_T479A_ = *P* > 0.05, M18_WT_ versus M18_T479D_ = *P* < 0.01, M18_T479D_ versus M18_T479A_ = *P* < 0.01). (**k**) Cumulative EPSC charge during 40 Hz train stimulation. (**l**) Quantification of the Readily Releasable Pool (RRP) based on back-extrapolation to the y-axis of a linear fit through the last 20 points of the cumulative EPSC charge (M18_WT_: n = 17; M18_T479A_: n = 17; or M18_T479D_: n = 19; *P* = 0.3). (**m**) Quantification of total cumulative charge transferred during 40 Hz train. (M18_WT_: n = 17; M18_T479A_: n = 17; or M18_T479D_: n = 19; *P* = 0.2). (Statistically tested using the Kruskal-Wallis test and Dunn’s multiple comparison test, M18_T473D_ not included).

Using whole-cell patch-clamp electrophysiology on single hippocampal neurons grown on glia islands (autapses), we investigated the effects of M18_T479A_ and M18_T479D_ on synaptic transmission and short-term plasticity using M18_WT_ as a positive control. Expression of M18_Y473D_, mimicking phosphorylation by Src, was previously shown to severely inhibit synaptic transmission^17^ and was used here as a negative control to test the successful conditional deletion of Munc18-1. Neurons expressing M18_T479A_ or M18_T479D_ showed a similar spontaneous mEPSC frequency (Fig. 3b) and amplitude (Fig. 3c) as neurons expressing M18_WT_. Similarly, evoked release was similar between M18_WT_, M18_T479A_, and M18_T479D_ (Fig. 3d,e). Paired pulse plasticity, evaluated with varying inter-stimulus intervals, was also similar among neurons expressing M18_T479A_, M18_T479D_, and M18_WT_ (Fig. 3f,g).

High-frequency train stimulation at 40 Hz showed similar depression of synaptic transmission in all three conditions (Fig. 3h,i). During high frequency stimulation, the readily releasable pool (RRP) of synaptic vesicles is depleted. Moreover, the estimated RRP size (based on back-extrapolation of the steady-state, Fig. 3l) and the total transferred charge (Fig. 3m) were similar in neurons expressing either of the Munc18-1 mutants compared to wildtype. However, neurons expressing M18_T479D_ showed significantly enhanced recovery after depletion during the 40 Hz train stimulation compared to neurons expressing M18_T479A_ or M18_WT_ (Fig. 3j). In conclusion, basic synaptic transmission, release probability, and RRP size are all unaltered by mimicking or preventing Dyrk1a-dependent phosphorylation of Munc18-1, but recovery of synaptic transmission after high frequency stimulation is enhanced by expression of M18_T479D_.

## Discussion

This study shows that T479 is among several phosphorylation sites in Munc18-1 that are constrained and can be phosphorylated *in vitro*. Despite other established strong effects of Munc18-1 phosphorylation^5,16–18,42,43^, T479 is not a major regulatory site under the conditions tested here. Synaptic transmission is maintained at wildtype level when mimicking or preventing Dyrk1a-dependent phosphorylation, except for recovery after intense stimulation. Mimicking the phosphorylated state of Munc18-1 increases synaptic transmission after intense stimulation under these conditions. Phosphorylation of Munc18-1 at different residues has consistently shown to be important for the regulation of synaptic transmission. Kinases such as PKC^14–16^, Src^17^ and ERK^18^ have a major impact on Munc18-1 function and synaptic transmission, regulating DAG-induced potentiation, SNARE complex formation, and degradation of Munc18-1, respectively. In the native protein, the ERK and Src phosphorylation sites, at serine (S) 241 and tyrosine (Y) 473, are in close proximity to the Dyrk1a phosphorylation site in the three dimensional crystal structure^44,45^, suggesting a regulatory hub. Thus, surprisingly, spontaneous, evoked synaptic transmission and most short-term plasticity parameters were unaltered by replacing wildtype Munc18-1 for one of the T479 mutants.

Park *et al*. hypothesized a Dyrk1a-dependent regulation of the interaction between Munc18-1 and syntaxin, despite T479 being localized further away from the interaction site^19^. T479 is located at the edge of domain 3b of Mun18-1. This region is not associated with binding to synaptobrevin/VAMP2 or syntaxin^44,45^, the canonical interactors of Munc18-1, which are essential for its function. A phosphorylation site that critically influences Munc18-1 function, the Src kinase phosphorylation site (Y473)^17^, is located close to T479. Furthermore, Y473 is a predicted binding site of synaptobrevin/VAMP2^17,45^. Despite being 6 amino acids away from Y473, mimicking or preventing phosphorylation at T479 does not have a strong regulatory effect on synaptic transmission and is therefore likely to be located away from where synaptobrevin/VAMP2 and Munc18-1 interact.

Using co-immunoprecipitation, we did not replicate the interaction between Munc18-1 and Dyrk1a reported previously^19^. In contrast to the full length Dyrk1a protein used in this study, we used a truncated form of Dyrk1a that comprises the n-terminal amino acid residues 1 to 499 including the kinase domain. The construct used was comprised primarily of the kinase domain and might therefore be missing important interaction sites in the C-terminal end that makes the interaction sufficiently stable to show using co-immunoprecipitation. However, Dyrk1a was not identified as an interactor of Munc18-1 in a previously conducted pulldown experiment either (data not shown). The interaction between Munc18-1 and Dyrk1a is therefore probably transient, sufficient for phosphorylation to occur but not stable enough to be picked up using pulldown experiments.

In neurons expressing the phosphorylation mimicking Munc18-1 mutant, high frequency stimulation was followed by increased EPSC size, consistent with either increased recovery of the RRP or increased release probability. This observation can be attributed to at least three mechanisms: the speed at which the RRP is refilled (endocytosis/re-use of vesicles^46,47^ or transport from the reserve pool to the RRP^48^); the number or vesicles at the active zone^49^; or the availability of Munc18-1 at the active zone^41,43^. It is difficult to distinguish which process contributes to the observed phenotype but increased synaptic levels of Munc18-1 after high frequency stimulation might be the most plausible seeing as the baseline Munc18-1 levels at the synapse were not affected. Moreover, acutely overexpressing Munc18-1 using Semliki virus shows a similar increase in EPSC size after a 40 Hz train stimulation^41^. Altered local levels of Munc18-1 at the synapse could change synaptic transmission by increasing the number of available fusion-ready vesicles. Dyrk1a-dependent phosphorylation is the first known phosphorylation site of Munc18-1 that does not regulate the role of Munc18-1 in synaptic transmission but mildly influences recovery after high frequency stimulation. In a neuronal network this could make the network more vulnerable or resistant after activity, which could be relevant in epilepsy.

Munc18-1 and Dyrk1a play an important role in brain development^6,29^. Both Munc18-1 and Dyrk1a haploinsufficiency cause seizures in human patients^8,11^ and mice^12,34^. For example, mice deficient in Munc18-1 as well as Dyrk1a display seizures and cognitive deficiencies^12,34^. *De novo* mutations in *DYRK1A* are found in 0.1–0.5% of people with autism^50^ and about 0.5% of people with syndromic forms of ID^51^. *STXBP1 and DYRK1A* have been associated with autism using whole exome sequencing^9^ and *DYRK1A* has been identified in several whole exome and target sequencing studies (see review^52^). *DYRK1A* and *STXBP1* associated disorders show symptomatic overlap. Core symptoms of *DYRK1A*-deficiency disorders include ID, microencephaly, developmental delay, seizures, and autistic feature (e.g., impairment in social interaction/communication and repetitive behaviours)^8,50,52,53^. and STXBP1 encephalopathy patients show severe ID, autistic features, and in 95% of the cases occurrence of epilepsy^11^. Hippocampal neurons expressing the phosphorylation mimicking or preventing mutants of Munc18-1 show normal synaptic transmission and only after high frequency stimulation does Dyk1a-dependent phosphorylation of Munc18-1 have a minor effect, increasing synaptic response. This stands in contrast to the similarities of the clinical phenotypes and they are therefore unlikely to be caused by the same mechanism and phosphorylation of Munc18-1 by Dyrk1a is not underlying the common symptoms observed in *DYRK1A* and *STXBP1* associated disorders.

## Methods

### Laboratory animals

Animal experiments were approved by the animal ethical committee of the VU University/VU University Medical Centre (license number: FGA 11-03) and are in accordance with Dutch governmental guidelines and regulations. Animals were housed and bred according to institutional, Dutch, and U.S. governmental guidelines. Munc18-1 deficient mice were generated as described previously^54^. *Munc18 null* mutant mice are stillborn and can be easily distinguished from wild-type or heterozygous littermates. E18 embryos were obtained by cesarean section of pregnant females from timed mating of heterozygous mice. *Munc18-1 lox* mice were generated as described previously^55^. Newborn *Munc18-1 lox* mice were obtained at P0-1 from a homozygous litter. Newborn P0-P1 pups from pregnant female Wistar rats were used for glia preparations.

### Constructs

Single amino acid substitutions on T479 in Munc18-1 were generated using Quikchange and verified by sequencing. The Munc18-1 constructs were connected with a T2 linker to Cre-EGFP and cloned into pLenti vectors, ensuring that expression levels of Cre-EGFP and Munc18-1 were similar. Viral particles were produced as described^56^. Transduction efficiencies of lentivirus containing Munc18-1 were assessed on HEK293T cells using a concentration range, and were taken into account when viruses were applied to neuronal cultures. For this purpose, Human embryo kidney 293 T (HEK293T) cells were infected with lentivirus 1 day after plating in DMEM containing 10% fetal calf serum (FCS; Gibco) and 1% penicillin/streptomycin (Gibco) and number of GFP positive cells were counted after 1 day.

### Immunoprecipitations

HEK293T cells were cultured in DMEM supplemented with 10% FCS, pen/strep(1:100) and non-essential amino acids (1:100; all Invitrogen) and plated in 6-wells dishes to 30% confluency on the day of transfection. Cells were transfected with Munc18-1 and myc tagged active Dyrk1a constructs (AA 1-499; Millipore) using calcium phosphate precipitation. The cells were washed 16 hr after transfection (DMEM). Immunoprecipitations were done 36 hours after transfections. Cells were lysed in IP-buffer (50 mM Tris-HCl pH 7.5, 1% Triton-X100, 1.5 mM MgCl_2,_ 5.0 mM EDTA, 100 mM NaCl). Lysates were centrifuged 10 min at 4 °C 16000 g and supernatants were collected. Antibodies against myc or munc18 were added to the supernatant. ProteinA Agarose beads were added (Vector Laboratories) and the samples were tumbled 2 hours at 4 degrees. After the immunoprecipitations were washed 5 times with IP-buffer, the samples were eluted from the beads with Laemmli sample buffer (2% SDS, 10% glycerol, 0.26 M B-mercaptoethanol, 60 mM Tris-HCl pH 6.8) and analysed with SDS-PAGE.

### Protein chemistry

Samples were run on a 9% SDS-PAGE gel and transferred to Immuno-Blot PVDF Membrane (bio-rad). Blots were stained for myc (Genetex) and Munc18-1 (cell signalling). Secondary antibodies were conjugated with Alkaline Phosphatase (Jackson lab) and Attophos (Promega) was used as substrate. The blots were imaged with the Image Reader FLA-5000 (Fuji)

### *In vitro* kinase assay

HEK293T cells were transfected with Munc18-1. 36 hours after transfection cells were lysed in 1x Laemmli sample buffer (2% SDS, 10% glycerol, 0.26 M B-mercaptoethanol, 60 mM Tris-HCl pH 6.8). After denaturing (boiling for 5 min) the samples were diluted 15x in IP-buffer (50 mM Tris-HCl pH 7.5, 1% Triton-X100, 1.5 mM MgCl_2,_ 5.0 mM EDTA, 100 mM NaCl). Munc18-1 was immune-precipitated with polyclonal Munc18-1 Antibody (cell signalling). ProteinA agarose beads were added and the samples were tumbled for 2 hours at 4 degrees. After 2 hours the precipitations were washed 2x with IP-buffer and 3x with 1x kinase buffer (New England Biolabs). Kinase mixes with either active Dyrk1a (Millipore) or ERK (Sigma Aldrich) were added to the samples (400 ng kinase for each sample). 25 μM [γ-32P]-ATP was added and samples were incubated at 37 °C for 30 minutes. The kinase reaction was stopped by adding 5ul 5x Laemmli sample buffer and samples were boiled for 5 minutes before analysing them with SDS-PAGE. The gels were imaged with the Image Reader FLA-5000 (Fuji).

### Dissociated neuronal cultures

Hippocampi from *Munc18-1 lox* mice were collected in ice-cold Hanks’ buffered salt solution (HBSS; Sigma) buffered with 7 mM HEPES (Invitrogen). After removal of the meninges, hippocampi were incubated in Hanks-HEPES containing 0.25% trypsin (from 10x stock, Invitrogen) for 20 minutes at 37 °C. After washing, neurons were triturated in DMEM, supplemented with 10% FCS, 1% nonessential amino acids (NAA) using a fire-polished Pasteur pipette and counted in a Fuchs-Rosenthal chamber. Neurons were plated in pre-warmed Neurobasal medium supplemented with 2% B-27, 1.8% HEPES, 0.25% glutamax, and 0.1% Pen/Strep (all Invitrogen) and infected with lentiviral particles encoding Munc18-1 variants after 3 days *in vitro* (DIV).

Hippocampal *Munc18-1 lox* neurons were plated on micro-islands of rat glia at a density of 1.5 k per well in a 12-well plate to achieve autaptic cultures. To generate micro-islands, glass coverslips (Menzel) were etched in 1 M HCl for at least 2 hours and neutralized with 1 M NaOH for maximum 1 hour, washed thoroughly with MiliQ water, and washed once with 70% ethanol. Coverslips were stored in 96% ethanol and coated with agarose type II-A (0.0015% in H_2_O, Sigma). Coating was done by spreading a thin layer of agarose solution (heated in a microwave and kept at 55 °C during use) with a cotton swab over the entire coverslip. Microdots were created using a custom-made rubber stamp (dot diameter 250 µm) to apply a solution consisting of 0.1 mg/ml poly-D-lysine (Sigma), 0.7 mg/ml rat tail collagen (BD Biosciences), and 10 mM acetic acid (Sigma) by stamping from a wet filter paper (3-mm cellulose chromatography paper, Whatman). Coverslips were UV-sterilized for 20 minutes before further use. Astrocytes were plated at 6–8 k/well in prewarmed DMEM (Invitrogen) supplemented with 10% FCS, 1% nonessential amino acids (NAA), and 1% penicillin/streptomycin (all Gobco).

### Electrophysiological recordings

Autaptic cultures of *Munc18-1 lox* neurons were grown for 12–15 days before electrophysiological measurements were performed. Whole-cell voltage-clamp recordings (V_m_ = −70 mV) were performed at room temperature. Borosilicate glass pipettes (2.5–4.5 mOhm) filled with 125 mM K^+^-gluconic acid, 10 mM NaCl, 4.6 mM MgCl, 4 mM K2-ATP, 15 mM creatine phosphate, 10 U/ml phosphocreatine kinase, and 1 mM EGTA (pH 7.3, 300 mOsmol). External solution was made up of 10 mM HEPES, 10 mM glucose, 140 mM NaCl, 2.4 mM KCl, 4 mM MgCl_2_, and 2 mM CaCl_2_ (pH 7.3, 300 mOsmol). Only excitatory neurons, identified by decay of the postsynaptic currents, were included. Recordings were acquired with an Axopatch 200B amplifier, Digidata 1440 A, and ClampX 10.2 software (Molecular Devices). After whole-cell mode was established, only cells with an access resistance of <15 mΩ and leak current of <500 pA were included in the analysis. Evoked EPSCs were elicited by a 1 ms long depolarization to 30 mV. RRP size was estimated using back-extrapolation by making a linear fit through the last 20 pulses of a 40 Hz stimulation train, where intracellular calcium levels are high and synchronous release has been mostly abolished. At this point newly-recruited fusion-capable vesicles are released immediately. This is considered to be a steady-state situation, at which a linear back-extrapolation procedure can be used to estimate the size of the initial RRP (y-axis intercept) corrected for vesicle recruitment. Offline analysis was performed using custom-written software routines in Matlab R2017b (Mathworks).

### Immunocytochemistry

Cover slips from the same 12-well plates used for electrophysiology were used each week to control Munc18-1 levels as well as assessment of morphology. Cultures were fixed with 3.7% formaldehyde (Electron Microscopy Sciences). After washing with PBS, cells were permeated with 0.5% Triton X-100 for 5 minutes and incubated in 2% normal goat serum and 0.1% Triton X-100 for 20 minutes to block non-specific binding. Cells were incubated for 2 hours at room temperature with a primary antibody mixture of polyclonal guinea pig anti-Synaptophysin-1 (1:1000, SYSY), polyclonal chicken anti-MAP2 (1:10,000, Abcam), and polyclonal rabbit anti-Munc18-1 (1:1000, described previously^57^) antibodies. After washing, cells were incubated for 2 hours at room temperature with secondary antibodies conjugated to Alexa dyes (1:1,000, Molecular Probes) and washed again. Coverslips were mounted with DABCO-Mowiol (Invitrogen).

### Microscopy

Images of single cells were acquired with a confocal microscope (LSM 510, Carl Zeiss) using a 40x oil immersion objective (NA = 1.3) with 0.7x zoom at 1024 × 1024 pixels and averaged over four scans. Neuronal morphology and protein levels were analysed using automated image analysis routine^58^. Whole coverslips images were acquired using a confocal microscope (Nikon A1 plus) with a 10x Plan Apo λ objective (NA = 0.45) at 1024 × 1024 pixels and averaged over two scans. Z-stacks were acquired with an interval of 3.7 µm, individual images were stitched together and maximally projected for analysis and display.

### Data analysis

Data is presented as mean values ± SEM, with *n* referring to the number of cells from each group. Statistical analysis was performed using GraphPad Prism 5 (GraphPad Software). Data was tested for normality with the Kolmogorov and Smirnov test. As the data was not normally distributed the nonparametric Kruskal-Wallis test and Dunn’s multiple comparison test were used. P-values below 0.05 are considered significant.

## Supplementary information

Supplementary Figure S1 - Original uncropped gels and blot.

## Data Availability

All the data generated during this work is presented in the paper. Datasets are available from the corresponding author upon reasonable request.

## References

[1] Collins MO (2005). Proteomic analysis of *in vivo* phosphorylated synaptic proteins. J. Biol. Chem..

[2] Huttlin EL (2010). A tissue-specific atlas of mouse protein phosphorylation and expression. Cell.

[3] Munton RP (2007). Qualitative and Quantitative Analyses of Protein Phosphorylation in Naive and Stimulated Mouse Synaptosomal Preparations. Mol. Cell. Proteomics.

[4] Tweedie-Cullen RY, Reck JM, Mansuy IM (2009). Comprehensive mapping of post-translational modifications on synaptic, nuclear, and histone proteins in the adult mouse brain. J. Proteome Res..

[5] de Jong AP, Verhage M (2009). Presynaptic signal transduction pathways that modulate synaptic transmission. Current Opinion in Neurobiology.

[6] Verhage M (2000). Synaptic assembly of the brain in the absence of neurotransmitter secretion. Science (80-.)..

[7] Santos TC, Wierda K, Broeke JH, Toonen RF, Verhage M (2017). Early Golgi Abnormalities and Neurodegeneration upon Loss of Presynaptic Proteins Munc18-1, Syntaxin-1, or SNAP-25. J. Neurosci..

[8] Courcet JB (2012). The DYRK1A gene is a cause of syndromic intellectual disability with severe microcephaly and epilepsy. J. Med. Genet..

[9] De Rubeis S (2014). Synaptic, transcriptional and chromatin genes disrupted in autism. Nature.

[10] Saitsu H (2008). De novo mutations in the gene encoding STXBP1 (MUNC18-1) cause early infantile epileptic encephalopathy. Nat. Genet..

[11] Stamberger, H. *et al*. STXBP1 encephalopathy A neurodevelopmental disorder including epilepsy. 1–10 (2016). 10.1212/WNL.0000000000002457 26865513

[12] Kovačević J (2018). Protein instability, haploinsufficiency, and cortical hyper-excitability underlie STXBP1 encephalopathy. Brain.

[13] Hornbeck PV (2015). PhosphoSitePlus, 2014: Mutations, PTMs and recalibrations. Nucleic Acids Res..

[14] Barclay JW (2003). Phosphorylation of Munc18 by protein kinase C regulates the kinetics of exocytosis. J. Biol. Chem..

[15] Genç Ö, Kochubey O, Toonen RF, Verhage M, Schneggenburger R (2014). Munc18-1 is a dynamically regulated PKC target during short-term enhancement of transmitter release. Elife.

[16] Wierda KDB, Toonen RFG, de Wit H, Brussaard AB, Verhage M (2007). Interdependence of PKC-Dependent and PKC-Independent Pathways for Presynaptic Plasticity. Neuron.

[17] Meijer M (2017). Tyrosine phosphorylation of Munc18‐1 inhibits synaptic transmission by preventing SNARE assembly. EMBO J..

[18] Schmitz SK (2016). Presynaptic inhibition upon CB1 or mGlu2/3 receptor activation requires ERK/MAPK phosphorylation of Munc18‐1. EMBO J..

[19] Park JH, Jung MS, Kim YS, Song WJ, Chung SH (2012). Phosphorylation of Munc18-1 by Dyrk1A regulates its interaction with Syntaxin 1 and X11α. J. Neurochem..

[20] Aranda S, Laguna A, de la Luna S (2011). DYRK family of protein kinases: evolutionary relationships, biochemical properties, and functional roles. FASEB J..

[21] Martí E (2003). Dyrk1A expression pattern supports specific roles of this kinase in the adult central nervous system. Brain Res..

[22] Aranda S, Alvarez M, Turro S, Laguna A, de la Luna S (2008). Sprouty2-Mediated Inhibition of Fibroblast Growth Factor Signaling Is Modulated by the Protein Kinase DYRK1A. Mol. Cell. Biol..

[23] Wegiel J (2004). Cell type- and brain structure-specific patterns of distribution of minibrain kinase in human brain. Brain Res..

[24] Hämmerle B (2003). Expression patterns and subcellular localization of the Down syndrome candidate protein MNB/DYRK1A suggest a role in late neuronal differentiation. Eur. J. Neurosci..

[25] Soppa U (2014). The down syndrome-related protein kinase DYRK1A phosphorylates p27Kip1and cyclin D1 and induces cell cycle exit and neuronal differentiation. Cell Cycle.

[26] Kurabayashi N, Sanada K (2013). Increased dosage of DYRK1A and DSCR1 delays neuronal differentiation in neocortical progenitor cells. Genes Dev..

[27] Guedj F (2012). DYRK1A: A master regulatory protein controlling brain growth. Neurobiol. Dis..

[28] Dang T (2018). Autism-associated Dyrk1a truncation mutants impair neuronal dendritic and spine growth and interfere with postnatal cortical development. Mol. Psychiatry.

[29] Martinez De Lagran M (2012). Dyrk1A influences neuronal morphogenesis through regulation of cytoskeletal dynamics in mammalian cortical neurons. Cereb. Cortex.

[30] Wegiel J, Gong CX, Hwang YW (2011). The role of DYRK1A in neurodegenerative diseases. FEBS Journal.

[31] Yamamoto T (2011). Clinical manifestations of the deletion of Down syndrome critical region including DYRK1A and KCNJ6. Am. J. Med. Genet. Part A.

[32] Dowjat WK (2007). Trisomy-driven overexpression of DYRK1A kinase in the brain of subjects with Down syndrome. Neurosci. Lett..

[33] Park J, Song WJ, Chung KC (2009). Function and regulation of Dyrk1A: towards understanding Down syndrome. Cell. Mol. Life Sci..

[34] Raveau M, Shimohata A, Amano K, Miyamoto H, Yamakawa K (2018). DYRK1A-haploinsufficiency in mice causes autistic-like features and febrile seizures. Neurobiol. Dis..

[35] Fotaki V (2002). Dyrk1A haploinsufficiency affects viability and causes developmental delay and abnormal brain morphology in mice. Mol. Cell. Biol..

[36] Kang JE, Choi SA, Park JB, Chung KC (2005). Regulation of the proapoptotic activity of Huntingtin interacting protein 1 by Dyrk1 and caspase-3 in hippocampal neuroprogenitor cells. J. Neurosci. Res..

[37] Ballif BA, Carey GR, Sunyaev SR, Gygi SP (2008). Large-Scale Identification and Evolution Indexing of Tyrosine Phosphorylation Sites from Murine Brain. J. Proteome Res..

[38] Havrilla JM, Pedersen BS, Layer RM, Quinlan AR (2019). A map of constrained coding regions in the human genome. Nat. Genet..

[39] Sitz JH, Tigges M, Baumgärtel K, Khaspekov LG, Lutz B (2004). Dyrk1A Potentiates Steroid Hormone-Induced Transcription via the Chromatin Remodeling Factor Arip4. Mol. Cell. Biol..

[40] Guo X, Williams JG, Schug TT, Li X (2010). DYRK1A and DYRK3 promote cell survival through phosphorylation and activation of SIRT1. J. Biol. Chem..

[41] Toonen RFG (2006). Munc18-1 expression levels control synapse recovery by regulating readily releasable pool size. Proc. Natl. Acad. Sci..

[42] de Jong APH (2016). Phosphorylation of synaptotagmin-1 controls a post-priming step in PKC-dependent presynaptic plasticity. Proc. Natl. Acad. Sci..

[43] Cijsouw T (2014). Munc18-1 redistributes in nerve terminals in an activity- and PKC-dependent manner. J. Cell Biol..

[44] Misura KM, Scheller RH, Weis WI (2000). Three-dimensional structure of the neuronal-Sec. 1-syntaxin 1a complex. Nature.

[45] Baker RW (2015). A direct role for the Sec. 1/Munc18-family protein Vps33 as a template for SNARE assembly. Science (80-.)..

[46] Cheung G, Jupp OJ, Cousin MA (2010). Activity-Dependent Bulk Endocytosis and Clathrin-Dependent Endocytosis Replenish Specific Synaptic Vesicle Pools in Central Nerve Terminals. J. Neurosci..

[47] Kavalali ET (2007). Multiple vesicle recycling pathways in central synapses and their impact on neurotransmission. J. Physiol..

[48] Kuromi H, Kidokoro Y (2003). Two synaptic vesicle pools, vesicle recruitment and replenishment of pools at the Drosophila neuromuscular junction. J. Neurocytol..

[49] Gulyas-Kovacs A (2007). Munc18-1: Sequential Interactions with the Fusion Machinery Stimulate Vesicle Docking and Priming. J. Neurosci..

[50] van Bon BWM (2016). Disruptive de novo mutations of DYRK1A lead to a syndromic form of autism and ID. Mol. Psychiatry.

[51] Evers JMG (2017). Structural analysis of pathogenic mutations in the DYRK1A gene in patients with developmental disorders. Hum. Mol. Genet..

[52] Arbones ML, Thomazeau A, Nakano-Kobayashi A, Hagiwara M, Delabar JM (2019). DYRK1A and cognition: A lifelong relationship. Pharmacol. Ther..

[53] Van Bon BWM (2011). Intragenic deletion in DYRK1A leads to mental retardation and primary microcephaly. Clinical Genetics.

[54] De Vries, K. J. *et al*. Dynamics of munc18-1 phosphorylation/dephosphorylation in rat brain nerve terminals. *Eur. J. Neurosci*. **12**(1) 385–390 (2000). 10.1046/j.1460-9568.2000.00931.x 10651895

[55] Heeroma JH (2004). Trophic support delays but not prevent cell-intrinsic degeneration of neurons deficient for munc18-1. Eur. J. Neurosci..

[56] Naldini L (1996). *In Vivo* Gene Delivery and Stable Transduction of Nondividing Cells by a Lentiviral Vector. Science (80-.)..

[57] Vries KJD, Geijtenbeek A, Brian EC, Graan PNED, Ghijsen WEJM (2000). Dynamics of munc18-1 phosphorylation/dephosphorylation in rat brain nerve terminals. Neuroscience.

[58] Schmitz SK (2011). Automated analysis of neuronal morphology, synapse number and synaptic recruitment. J. Neurosci. Methods.

