# Supplementary material for: A Munc18-1 mutant mimicking phosphorylation by Down Syndrome-related kinase Dyrk1a supports normal synaptic transmission and promotes recovery after intense activity
Source: Sci Rep. 2020 Feb 21;10:3181. doi: 10.1038/s41598-020-59757-y (PMC7035266; doi:10.1038/s41598-020-59757-y)
Supplement: Supplementary file 1 — Supplementary Figure S1 - Original uncropped gels and blot. [file 41598_2020_59757_MOESM1_ESM.pdf]

Supplementary files

A Munc18-1 mutant mimicking phosphorylation by Down Syndrome-related kinase Dyrk1a supports normal synaptic transmission and promotes recovery after intense activity

Jessica Classen[1], Ingrid Saarloos[1], Marieke Meijer[1], Patrick F Sullivan[2], Matthijs Verhage[1#]

[1] Department of Functional Genomics, Center for Neurogenomics and Cognitive Research, VU University, 1081 HV Amsterdam, The Netherlands

[2] Department of Medical Epidemiology and Biostatistics, Karolinska Institutet, Nobels väg 12A, PO Box 281, 171 77 Stockholm, Sweden; and Departments of Genetics and Psychiatry, University of North Carolina at Chapel Hill, Chapel Hill, North Carolina, USA

[#] To whom correspondence should be addressed: Matthijs Verhage, Center for Neurogenomics and Cognitive Research, VU University, De Boelelaan 1087, 1081 HV Amsterdam, The Netherlands. Email: matthijs@cncr.vu.nl

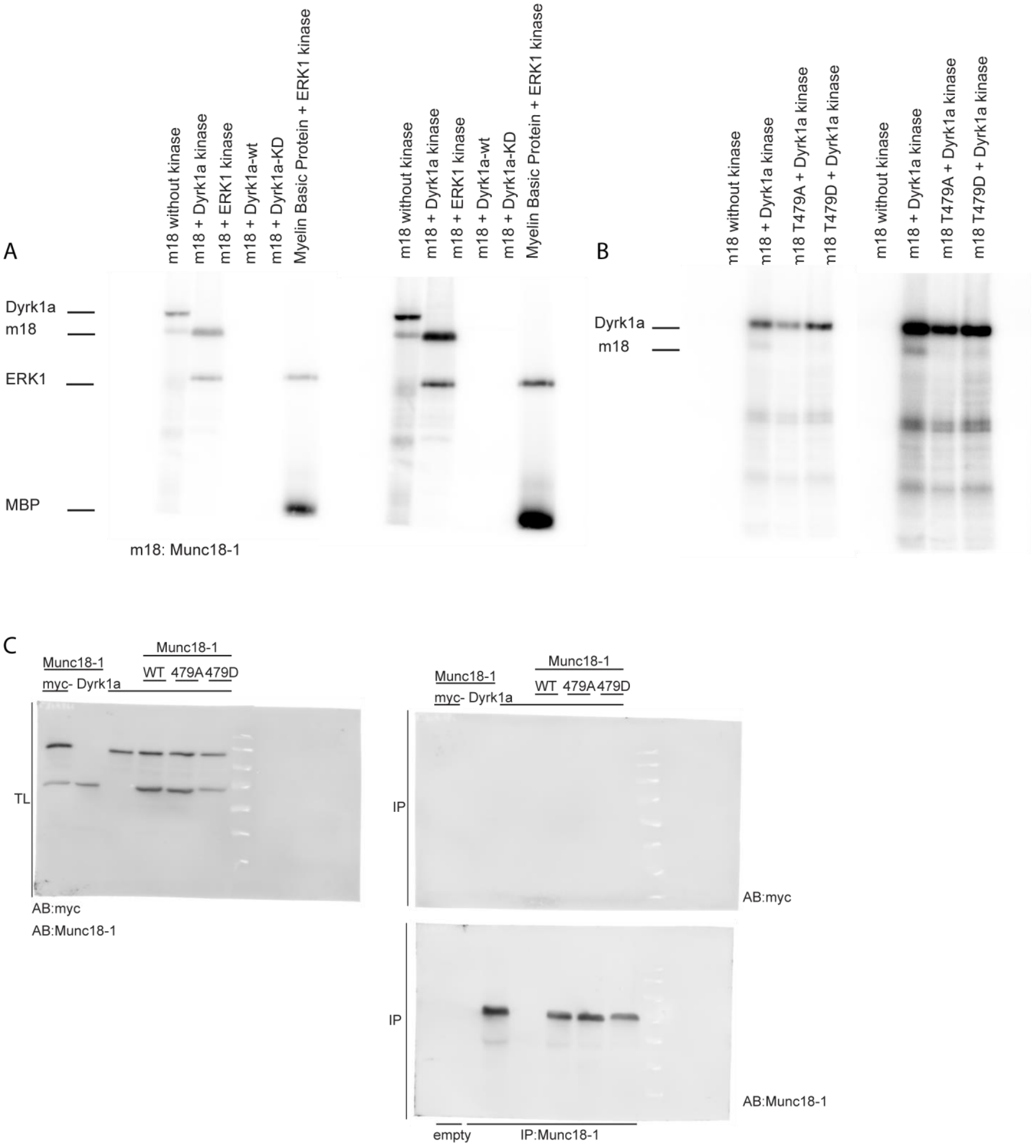

Supplementary Figure S1 – Original uncropped gels and blots.
Original gels from Fig 1b (A) and Fig 1c (B). Original blots from Fig 1d (C).

Supplementary Table S1: STXBP1 phosphorylation sites and mutation intolerance

genome build GRCh37 (hg19)

ccrpct = conserved coding region percentile

gene	bisque position	Source Position	Source Residue	chrom	base pair	ccrpct
STXBP1	T107	107	T	chr9	130422381	
STXBP1	T107	107	T	chr9	130422382	
STXBP1	T107	107	T	chr9	130422383	
STXBP1	S142	142	S	chr9	130423479	98.19
STXBP1	S142	142	S	chr9	130423480	98.19
STXBP1	S142	142	S	chr9	130423481	98.19
STXBP1	Y145	145	Y	chr9	130425487	98.19
STXBP1	Y145	145	Y	chr9	130425488	98.19
STXBP1	Y145	145	Y	chr9	130425489	98.19
STXBP1	S146	146	S	chr9	130425490	98.19
STXBP1	S146	146	S	chr9	130425491	98.19
STXBP1	S146	146	S	chr9	130425492	98.19
STXBP1	S241	241	S	chr9	130428502	99.89
STXBP1	S241	241	S	chr9	130428503	99.89
STXBP1	S241	241	S	chr9	130428504	99.89
STXBP1	S306	306	S	chr9	130432190	88.6
STXBP1	S306	306	S	chr9	130432191	88.6
STXBP1	S306	306	S	chr9	130432192	88.6
STXBP1	S313	313	S	chr9	130432211	88.6
STXBP1	S313	313	S	chr9	130432212	88.6
STXBP1	S313	313	S	chr9	130432213	88.6
STXBP1	S345	345	S	chr9	130435463	96.03
STXBP1	S345	345	S	chr9	130435464	96.03
STXBP1	S345	345	S	chr9	130435465	96.03
STXBP1	T346	346	T	chr9	130435466	96.03
STXBP1	T346	346	T	chr9	130435467	96.03
STXBP1	T346	346	T	chr9	130435468	96.03
STXBP1	Y473	473	Y	chr9	130440767	98.57
STXBP1	Y473	473	Y	chr9	130440768	98.57
STXBP1	Y473	473	Y	chr9	130440769	98.57
STXBP1	T479	479	T	chr9	130440785	98.57
STXBP1	T479	479	T	chr9	130440786	98.57
STXBP1	T479	479	T	chr9	130440787	98.57
STXBP1	S509	509	S	chr9	130442499	
STXBP1	S509	509	S	chr9	130442500	
STXBP1	S509	509	S	chr9	130442501	
STXBP1	S511	511	S	chr9	130442505	
STXBP1	S511	511	S	chr9	130442506	

STXBP1	S511	511	S	chr9	130442507	
STXBP1	T512	512	T	chr9	130442508	
STXBP1	T512	512	T	chr9	130442509	
STXBP1	T512	512	T	chr9	130442510	
STXBP1	S516	516	S	chr9	130442520	
STXBP1	S516	516	S	chr9	130442521	
STXBP1	S516	516	S	chr9	130444685	
STXBP1	T574	574	T	chr9	130453071	99.26
STXBP1	T574	574	T	chr9	130453072	99.26
STXBP1	T574	574	T	chr9	130453073	99.26
STXBP1	E590	590	E	chr9	130453119	99.26
STXBP1	E590	590	E	chr9	130453120	99.26
STXBP1	E590	590	E	chr9	130453121	99.26
STXBP1	S594	594	S	chr9	130453131	
STXBP1	S594	594	S	chr9	130453132	
STXBP1	S594	594	S	chr9	130453133	

Supplementary Table S2: Overlap of T479 surrounding area with mutation intolerant area

c0, c1, c2 are the coordinates of a constrained coding region (GRCh38 hg38)

OverlapCCR = True is if region of interest overlaps with a constrained coding region (CCR)

gene	site	chr	start	end	c0	c1	c2	OverlapsCCR
STXBP1	T479	9	127678506	127678508	chr9	127678456	127678532	TRUE
STXBP1	Region surrounding T479 (45bp)	9	127678485	127678529	chr9	127678456	127678532	TRUE
